# A Method for Expanding Mass Range on a Multi-Turn Time-of-Flight Mass Spectrometer by a Lap Superimposed Spectrum

**DOI:** 10.5702/massspectrometry.A0098

**Published:** 2021-10-14

**Authors:** Toshinobu Hondo, Michisato Toyoda

**Affiliations:** 1 MS-Cheminformatics LLC, Toin, Mie 511–0231, Japan; 2 Project Research Center for Fundamental Sciences, Graduate School of Science, Osaka University, 1–1 Machikaneyama, Toyonaka, Osaka 560–0043, Japan

**Keywords:** time-of-flight mass spectrometer, multi-turn, lap-difference, peak assign algorithm

## Abstract

A time-of-flight mass spectrometer that uses a closed-orbit flight path can achieve a high mass resolving power and a high mass accuracy with a small instrument footprint. It has long been known that a drawback to a closed flight path is an obtained spectrum may contain peaks by ions at a different number of laps. A lower *m*/*z* ion may overtake higher *m*/*z* ions, resulting in the peak being superimposed on an acquired mass spectrum; therefore, such a mass bandwidth of the analyzer is limited to a narrow range given the current situation. However, recent research has documented a solution to the problem based on careful study of the equation of motion of an ion in a closed-path analyzer. All of the ions in the analyzer remain in motion in orbit by the nature of the closed flight path, thus resulting in a superimposed spectrum with the width of the orbital period of the highest mass in the sample matrix, which contains several different lap numbers. When target ions for the sample are known in advance, the time-of-flight for a given *m*/*z* can be determined regardless of the lap number under given analyzer conditions, and peak assignment can be self-validated by comparison to a mass spectrum acquired at a different lap condition. Furthermore, the *m*/*z* value for an unknown ion can also be determined by comparing time-of-flight values on spectra acquired at different lap conditions.

## INTRODUCTION

A closed-orbit flight path is an effective way to achieve high mass resolving power on a time-of-flight (TOF) mass spectrometer while keeping the instrument footprint small. There are two such types of mass spectrometers that have been introduced in the past few decades, namely, multi-reflectron and multi-turn mass spectrometers. Wollnik and Przewloka developed a multi-reflection TOF mass spectrometer,^1)^ which contains multiple ion mirrors within a 70 cm length flight tube. Schury *et al.* achieved a mass resolving power of approximately 150,000 using a pair of ion-mirror multi-reflection mass spectrometers^2)^ designed for high precision mass measurements of short-lived nuclei.

Toyoda *et al.*^3)^ introduced a multi-turn TOF mass spectrometer (MULTUM Linear plus) featuring a figure-eight ion orbit mass analyzer packaged within a 60 cm×70 cm×20 cm vacuum chamber. This instrument achieved a mass resolving power of 350,000 at *m*/*z* 28. A miniaturized multi-turn TOF mass spectrometer (MULTUM-S II)^4)^ was then introduced. The instrument was packaged into a 50 cm×57 cm×30 cm enclosure that included the vacuum pumps. The set up consists of ion injection and ejection sectors, which are switched to manage ion passage through the analyzer, and four electrostatic sectors that are located on the corners of the figure-eight orbit. In this analyzer, ions are initially in motion in the figure-eight orbit and are then ejected toward the detector after a given number of laps. This implies that the molecular identification (mass) accuracy can be verified in real-time by monitoring an analyte at two different laps and overlaying the spectra, as previously reported.^5–7)^

Both multi-reflection and multi-turn mass analyzers, which involve the use of a closed flight path, may cause a spectrum for ion peaks to be superimposed due to the lighter ion overtaking the heavier ions. This has been discussed for the multi-reflectron analyzer in the literature and is referred to as the mass bandwidth.^2,8,9)^

**Figure 1:**
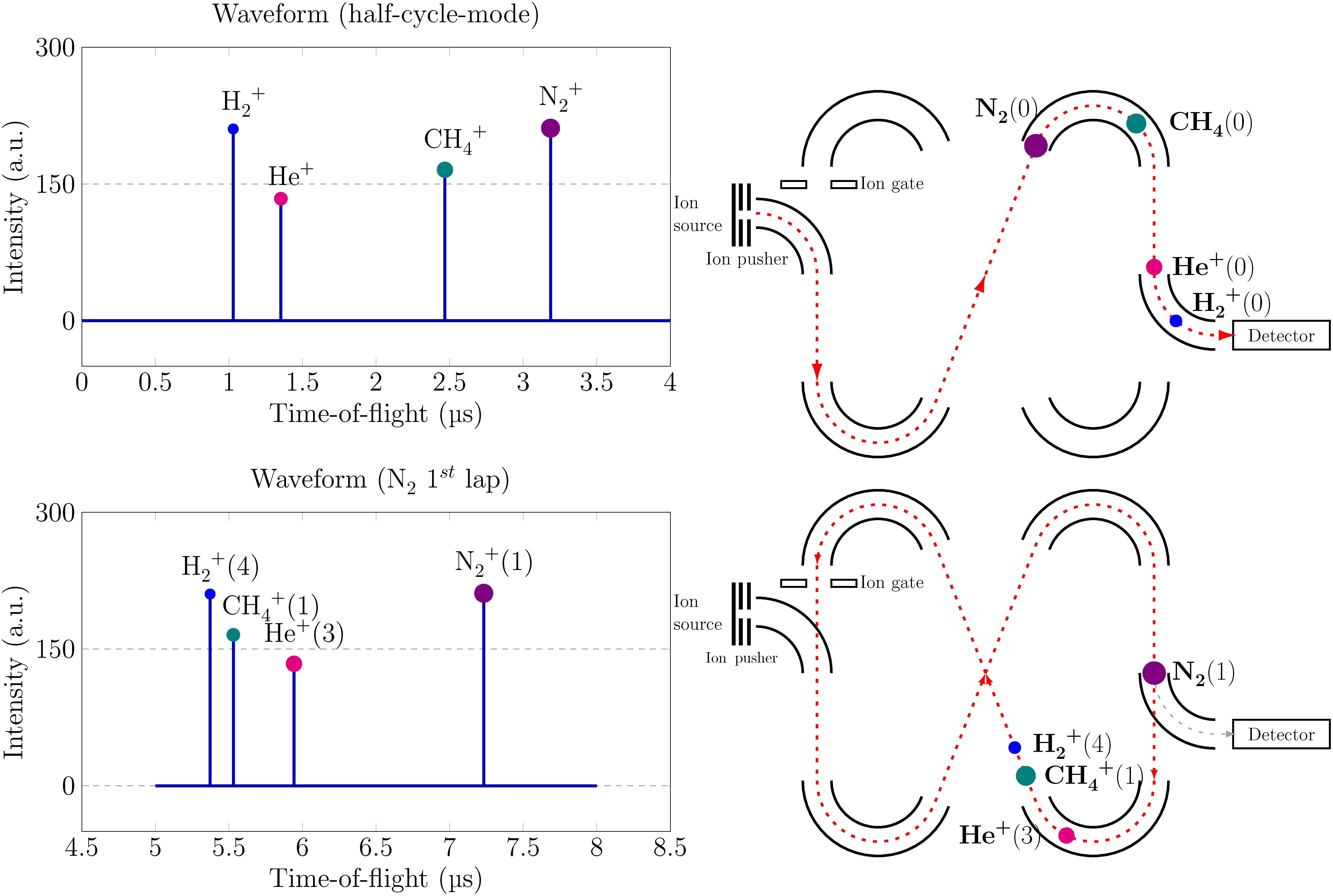
Fig. 1. Schematic representation of the motion of ions in the half-cycle mode (top), and first lap (bottom).

The ion peak superimposed by unmatched lap numbers is the result of an analyte in the sample matrix whose ion is faster or slower than the orbital period of the target analyte ion at a given condition. Figure 1 shows a schematic representation of a figure-eight orbit and the relative ion positions. Figure 1 top represents an ideal sample matrix containing H_2_, He, CH_4_, and N_2_ ions at the half-cycle mode. No superimposed ion peaks can be observed on the half-cycle mode because no closed path is used. In contrast, as shown in the bottom of Fig. 1, when ion laps using a closed path, at a time when an N_2_ ion comes back to the ejection sector entrance on the first lap, the fourth lap of the H_2_^+^ and the third lap of He^+^ will appear 1.86 and 1.28 μs ahead of the N_2_ ion, respectively. This will create a lap-superimposed spectrum consisting of several lap numbers, where the number of laps for each ion is determined by its own orbital period and the time to ejecting ions. When an obtained spectrum has ions superimposed from different lap numbers, the mass for each peak cannot be uniquely assigned and a distributed list of mass candidates can be obtained.

Such a superimposition can be eliminated by excluding the ions that have a flight time outside the N_2_^+^ orbital period during the initial lap cycle. For this reason, MULTUM-S II is equipped with an ion gate that can exclude ions from the figure-eight orbit when it is set to on. Therefore, the mass bandwidth for the MULTUM-S II is no more than the orbital period of the highest mass in the sample matrix, which is approximately 5 and 1 Da for 10 and 50 laps of an N_2_ ion, respectively.

Since the TOF for a given mass on this mass spectrometer accurately follows the TOF equation,^5)^ the ion gate and ejection sector switch timing can be determined by a simple calculation.^6,10,11)^

In the case where an ion gate is not used, all of the ions that are injected into the analyzer at an ion push event remain in motion in the figure-eight orbit until the ejection sector is opened. The obtained lap-superimposed TOF spectrum represents a fingerprint of all the ions within a time duration that corresponds to an orbital period for the highest mass ion. As an example, consider a sample mixture of CH_4_ (*m*/*z* 16.031), C_2_H_4_ (*m*/*z* 28.031), O_2_ (*m*/*z* 31.989), and CO_2_ (*m*/*z* 43.989). In this case, all of the ions are shown in the order of their masses calculated from half-cycle mode data, where lap-superimposition does not occur. However, the order will change to C_2_H_4_^+^, O_2_^+^, CH_4_^+^, and CO_2_^+^ at the first lap of the CO_2_^+^ spectrum.

This present study reports on expanding the previously reported mass assignment algorithms^6)^ into a mass assignment for a lap-superimposed spectrum that consists of unmatched lap numbers of ions.

## EXPERIMENTAL

### Apparatus

The miniaturized multi-turn TOF mass spectrometer infiTOF-UHV (infiTOF) (MSI.Tokyo, Inc., Tokyo, Japan), which was derived from the MULTUM-S II multi-turn TOF spectrometer,^4)^ was used with previously reported in-house modifications.^5,10)^ A MIGHTION^12)^ (Hamamatsu Photonics K.K., Hamamatsu, Japan) was used as an ion detector. The microchannel plate (MCP)-in potential was set to −2.6 kV; MCP voltage was set to −600 V. A voltage of 350 V was applied to the avalanche diode.

The detector signal was passed through a C11184 pre-amplifier (Hamamatsu Photonics K.K., Hamamatsu, Japan) and into an Acqiris U5303A 1 GSs^−1^ high-speed digitizer (Acqiris, Geneva, Switzerland). Data acquisition was performed on a dual Intel® 8-core Xeon® processor PC with a Linux (Debian 9.14) operating system using the open-source “QtPlatz” (https://github.com/qtplatz) software and a plugin for the infiTOF system.

A standard gas consisting of 279 ppbv N_2_O, 1.47 ppmv CH_4_, and 421 ppmv CO_2_ in N_2_ (Takachiho Chemical Industrial, Tokyo, Japan) was used as the model sample matrix. The model sample gas was introduced into the electron ionization (EI) chamber using an inactivated fused silica capillary with a length of 10 m and an inner diameter of 0.1 mm. The ionization chamber pressure was maintained at 8×10^−4^ Pa.

### Determination of TOF Equation

Equation 1 is the TOF equation^13)^ arranged for the infiTOF, where *t* is the TOF, *L_k_* is the half-cycle length, *L_c_* is the figure-eight orbit length, *n* is the number of laps, *V_acc_* is the acceleration voltage, *m* is the mass of the ion, *z* is the charge state of the ion, *K_amc_* is the atomic mass constant, *e* is the elementary charge, and *t*_0_ is the instrumental time-delay. 
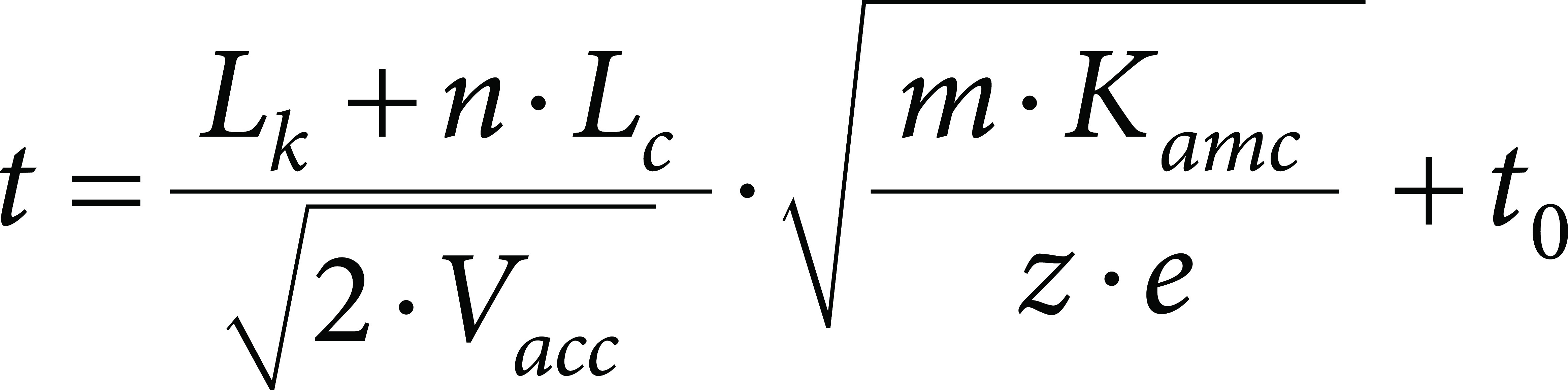
(1)

The value for *L_k_* was determined experimentally from the TOF of an ion measured at two different lap numbers, such as: 
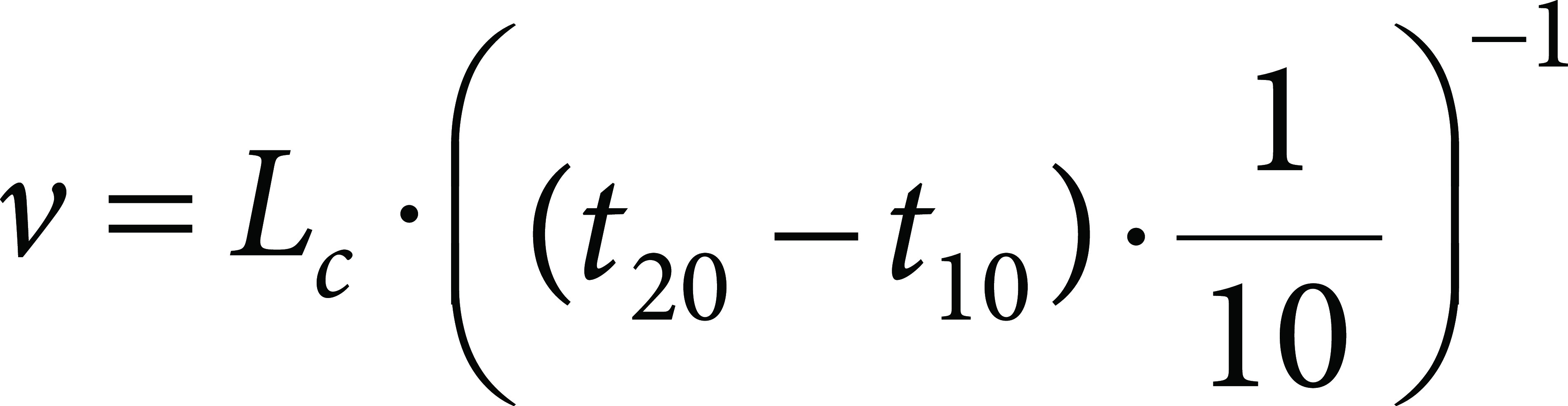
(2)
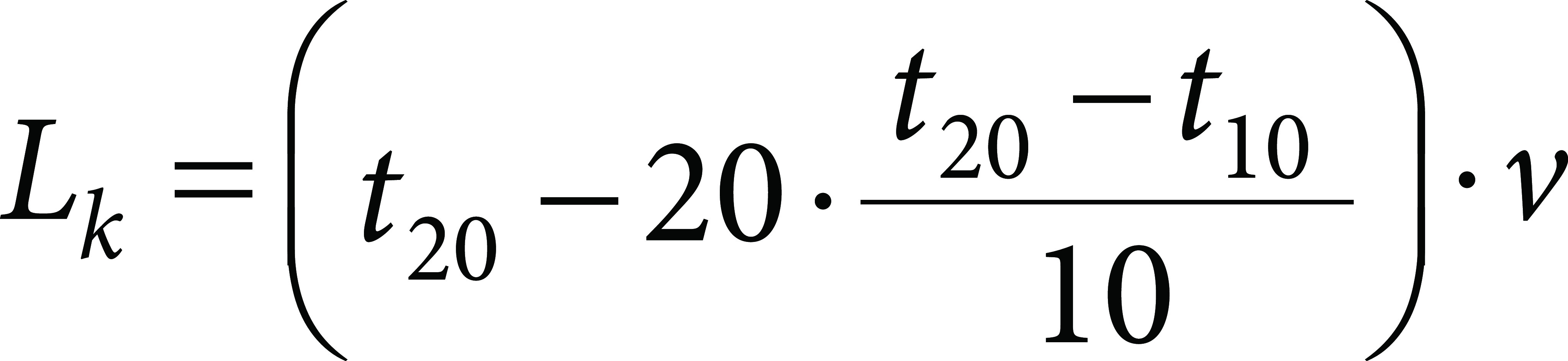
(3) where *v* is the velocity of CO_2_^+^, and *t*_10_ and *t*_20_ are the observed TOF for 10 and 20 laps of CO_2_^+^, respectively. The *L_k_* value was carefully determined using 20, 30, and 50 laps of CH_4_^+^, N_2_^+^, Ar^+^, and Xe^+^ using the least mean square method. To simplicity, we discuss the ions, which are charge state one hereafter.

Once *L_k_* was determined, *V_acc_* and *t*_0_ were calculated from the TOF obtained from different lap numbers of CO_2_^+^ using least mean squares. By using the TOF from 10, 20, and 30 laps of CO_2_^+^, the estimated value for *V_acc_* was 3893.22 V, and *t*_0_ was 0.240 μs. 
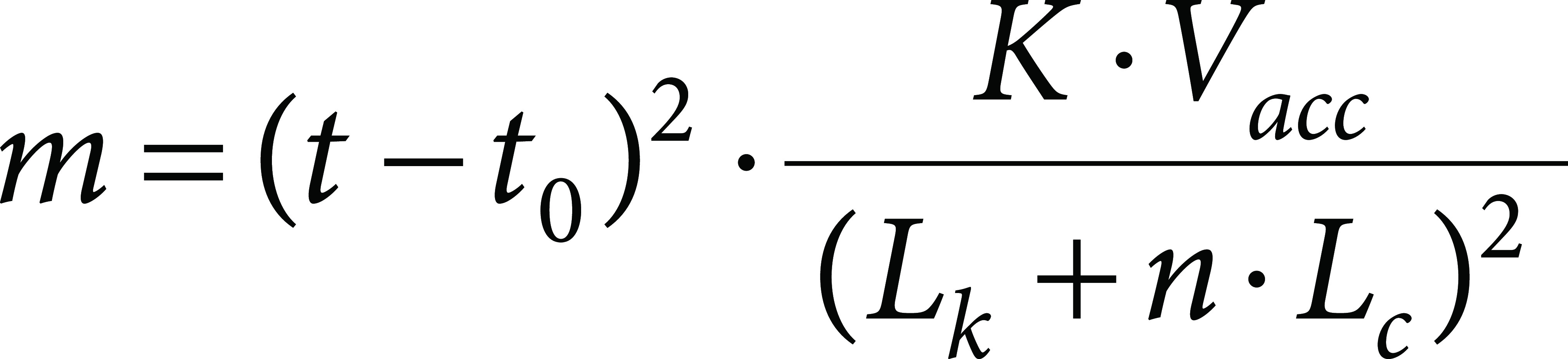
(4) where *K* is 
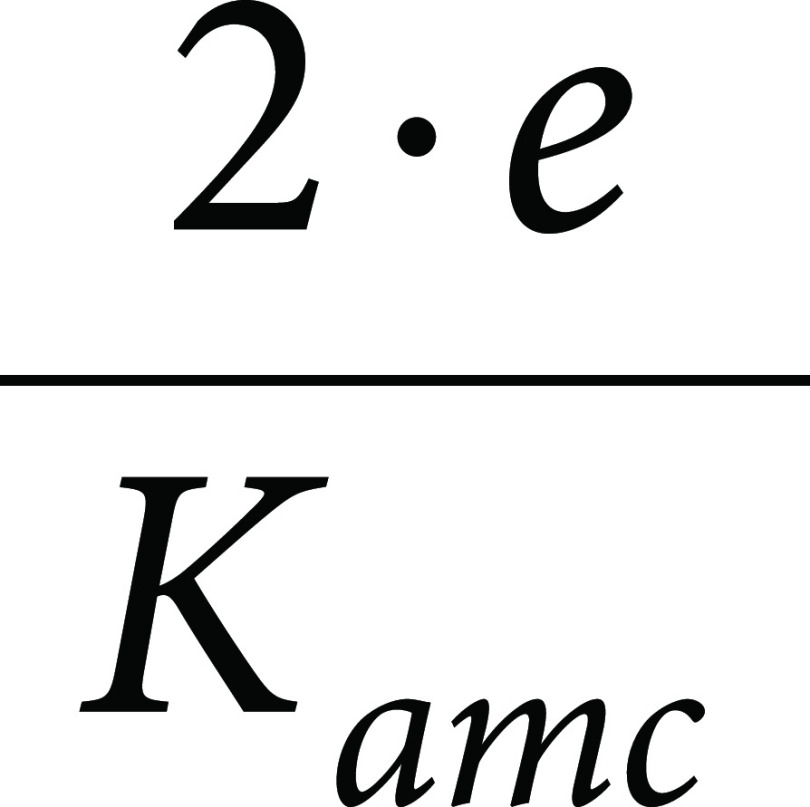


By using Eqs. 1 and 4, the TOF for a given mass and lap number (*n*), as well as *m*/*z* for a given TOF and lap number (*n*), can be calculated.

### Mass assignment algorithm for a known target ion

Target analytes that may be present in the sample matrix are known in advance in many cases. In such a case, a list of chemical formulae is generated, and a matching ion peak on the spectrum can be found from a single lap-superimposed mass spectrum, which contains several different lap numbers of ions. Assume that a mass spectrum was acquired for CO_2_^+^ at 24 laps and that ions from different laps were present. A CO_2_^+^ peak should appear at a TOF of 125.64 μs on the spectrum according to Eq. 1 and the previously determined *V_acc_* and *t*_0_ values. Under these conditions, the lap number for any given *m*/*z* can be uniquely determined as follows: calculate the TOF using Eq. 1 for a given *m*/*z* and ejection sector open timing. The flight length *L* for a given *m*/*z* and *t* can be calculated using Eq. 3. 
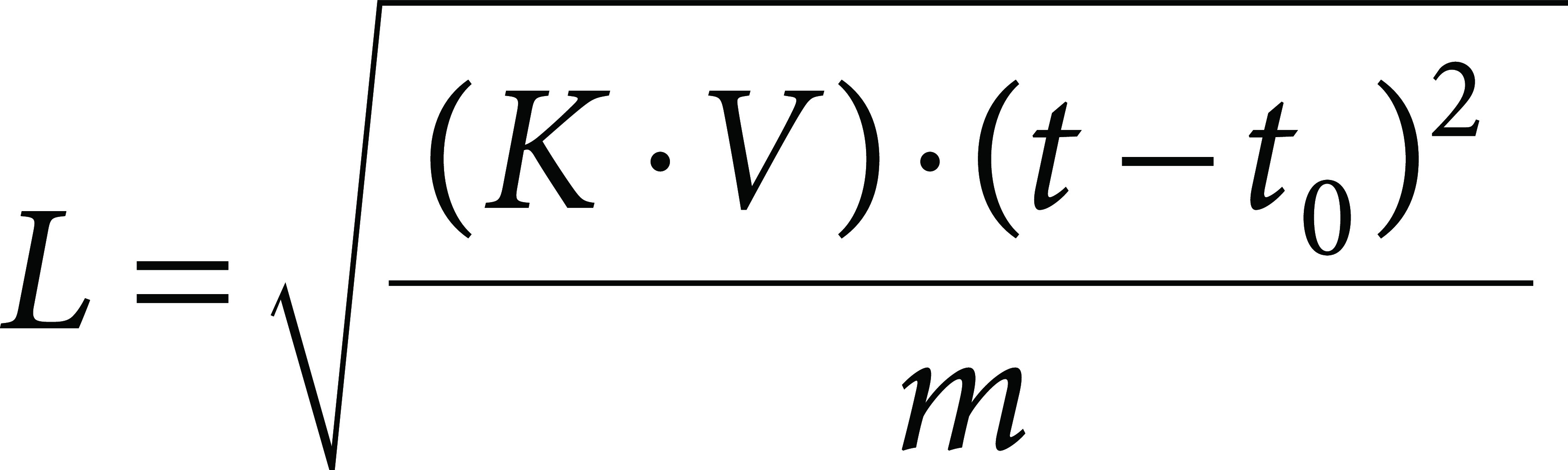
(5)Since *t* is a function of *n* as shown in Eq. 1, *L* is also a function of *n*. Accordingly, the number of laps can be determined as: 
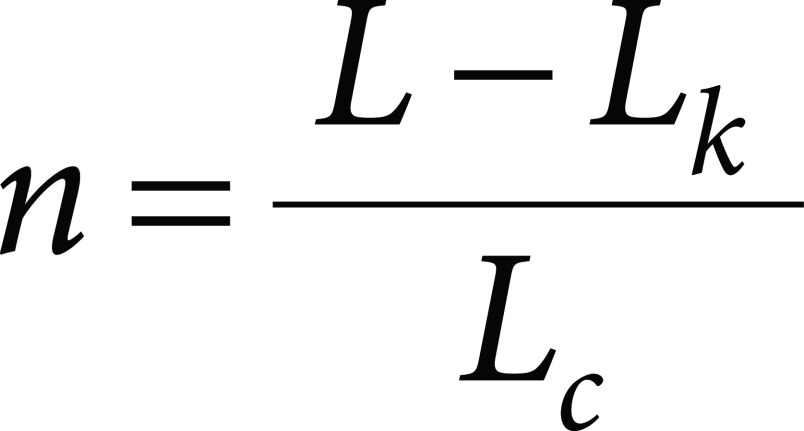
(6)

The obtained value *n* is the lap number for a given *m*/*z* that appeared at time *t* under a given ejection sector open timing. Of course, ejection sector timing depends on the length from the ejection sector to the detector, which is about 1/10 of the orbital length. The ion changes its lap number only when ejection sector timing moves over the ion’s orbital period threshold; therefore, the use of the length from the design schematics is sufficiently accurate.

The ejection sector open timing (123.11 μs) is automatically set for about half of the CO_2_^+^ orbital period earlier than the TOF of 24 laps in advance by the control software. Any ion flying faster enough to overtake CO_2_^+^ by 123.11 μs of duration will fly more than 25 laps. For example, from Eq. 1, O_2_^+^ will fly 28 laps, and the TOF should be 124.477 μs in the case of CO_2_^+^ 24 laps. This results in a spectrum in which 24 laps for CO_2_^+^ and 28 laps for O_2_^+^ are superimposed on a spectrum of only about 5.07 μs width. An *m*/*z* 43.176 can also be obtained for the TOF 124.477 μs peak with an assumption that the ion flown 24 laps from Eq. 4. Such an *m*/*z* calculated from an assumed lap number for all TOF peaks is called the “apparent *m*/*z*.” When we have a list of ions that are supposed to exist, both TOF and the “apparent *m*/*z*” can be calculated in advance and can be monitored in real-time during spectral acquisition. This approach is handy for many applications where measurements need to be compared against a list of chemical formulae of interest. One possible concern regarding this method is the possibility of a false peak assignment, however, this can be eliminated by acquiring spectra at multiple lap numbers. For example, C_2_H_4_^+^ (30 laps) and Ar^+^ (25 laps) have TOF values of 124.631 μs and 124.596 μs, respectively, with a calculated apparent *m*/*z* of 43.284 and 43.259, respectively, at 24 laps CO_2_ condition, which was the difference of 35 ns (24 mDa). However, both ions appear at 136.775 μs and 134.262 μs (apparent *m*/*z* of 44.636 and 43.009), which gives a difference of 2.51 μs (1.63 Da).

Therefore, lap number and peak assignment can be self-validated by acquiring at least two spectra using different lap conditions in sequence.

### Mass assignment algorithm for unknown ion

The data acquisition software automatically sets the analyzer ejection sector timing from user-set parameters, a pair of *m*/*z* (chemical formulae with charge), and a lap number for an ion of interest. The issue of whether a specified ion exists or not is irrelevant, but necessary for tentatively assigning masses on the spectrum. An acquired mass spectrum has assigned masses (“apparent *m*/*z*”) calculated by a user-specified ion and the lap number.

An *m*/*z* for an unknown ion can be determined if a pair of peaks from two different lap number conditions can be found. As an example, consider an ion at *n*_1_ and *n*_2_ laps analyzer conditions, where *n*_1_ and *n*_2_ are determined by the highest *m*/*z* in the sample. Comparing two initially assigned masses in both spectra, and finding ions where masses are not matched on both spectra, the lap number for those ions are neither *n*_1_ nor *n*_2_. By using Eq. 4 for several *n* values starting with *n*_1_ and *n*_2_ towards a higher lap number, and find a pair of lap numbers where the calculated *m*/*z* match. For example, assuming a pair of spectra acquired by two arbitrary analyzer conditions, which only differ in the timing of the ejection sector for different laps of the ion, the pair of TOF values for this unknown ion can be expressed as follows: 
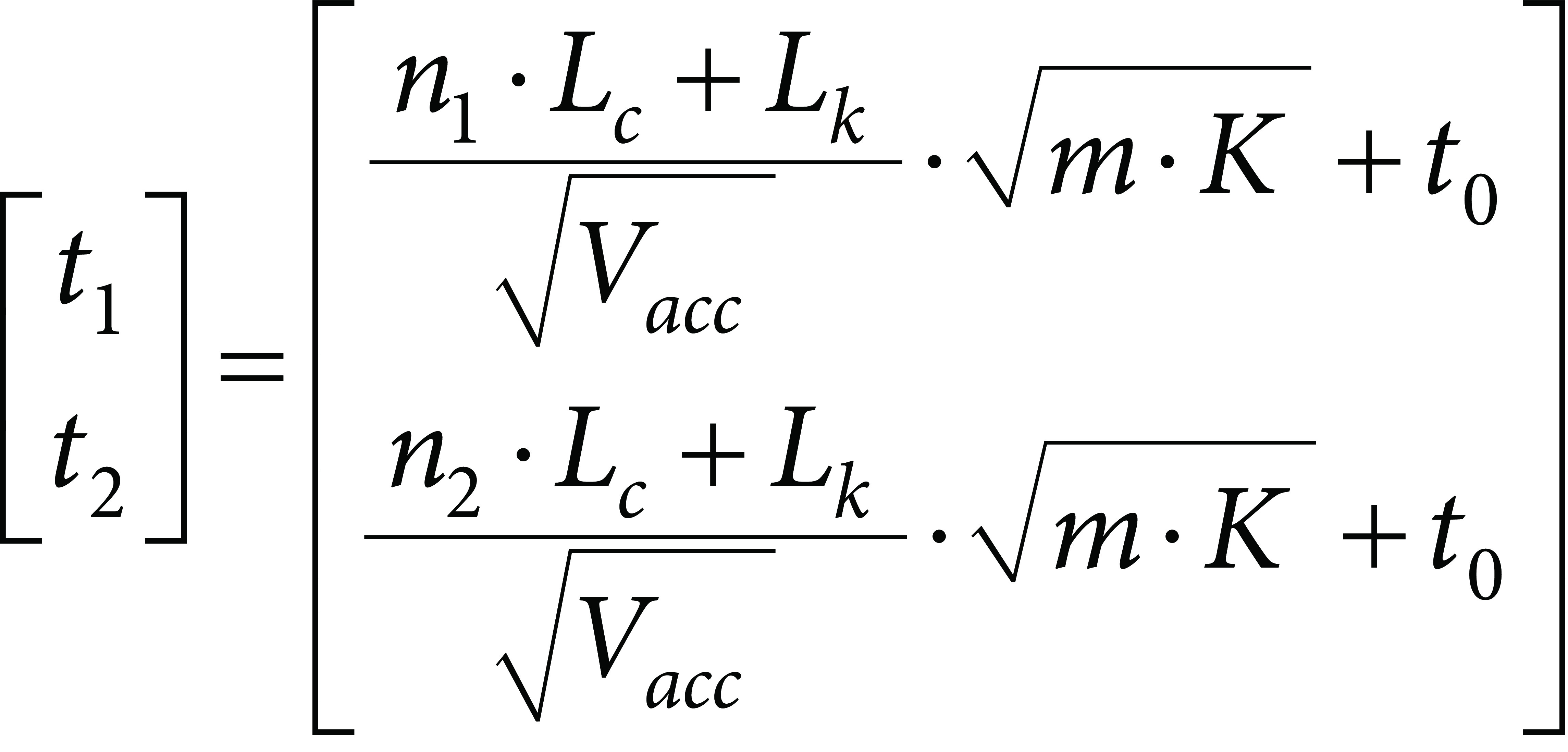
(7) where *K* is 2·(*e*/*K_amc_*), *n*_1_ and *n*_2_ are the unknown lap numbers, and *m* is *m*/*z* to be determined. Although Eq. 7 cannot be analytically solved for *m*, it can be determined computationally. The possible *m*/*z* values from the *n*_1_ condition spectrum can be expressed as: 
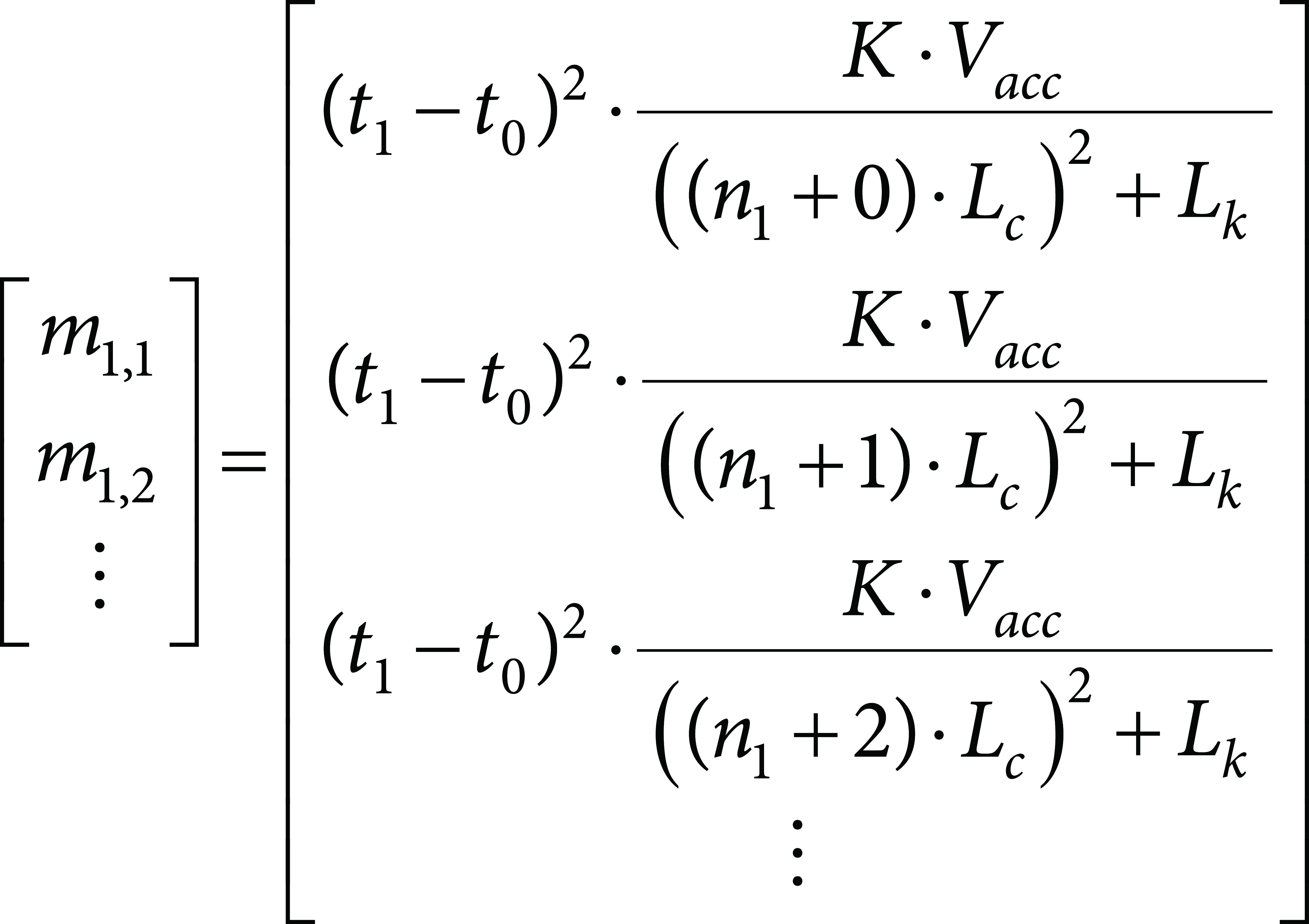
(8)

A sequence of *m*_1_*_,_*_1_, *m*_1_*_,_*_2_, . . . are the list of *m*/*z* candidates for the *n*_1_ condition, which can also be calculated for *n*_2_. A pair of sequences from the obtained TOF *t*_1_ and *t*_2_ can be solved to find a match. The numbers in the sequence are discrete because they are in *m*/*z* units for each lap period of the ion, which makes it easy to determine identical *m* values in the two sequences. In the case where the highest *m*/*z* in the sample is 600 and the analysis involves 20 laps, the ejection sector open timing should be 377.2 μs from the ion push event. Under such conditions, an ion such as *m*/*z* 14 will be detected after 132 laps. The lap numbers for all ions between *m*/*z* 14 and 600 can be found in a range of 20 to 132 laps (113 candidate masses in a sort order array). For a counterpart spectrum taken at 10 laps for *m*/*z* 600 that will be ejected at 189.93 μs, *m*/*z* 14 will fly 67 laps and has 58 candidate masses. Entering those candidate masses calculated by Eq. 4 into two vectors, the common value in two vectors can be found by using the “set intersections” algorithm.^14)^ The required computation to complete this algorithm to find a pair of matches from two vectors of 113 and 58 items is only 2·(113+58) comparisons.

The above scenario was generalized for lap number and *m*/*z* for a completely unknown ion. In practice, we can use a half-cycle mode spectrum as a counter spectrum of a pair, so that the *m*/*z* value for a peak is known to an accuracy of at least 10 mDa, which is nearly 100 times the accuracy compared to the *m*/*z* difference for a lap of H^+^ in 24 and 25 laps. The mass assigned by the half-cycle mode is always correct, in which the *n*_1_ is fixed to zero, then *n*_2_ can be quickly found from a high-resolution spectrum under longer flight path conditions. In this case, we can use a binary search algorithm^15)^ to find a match, which requires only 7 comparisons to find the matching *m*/*z* from the 113 candidates. Once *m*/*z* can be determined, the accuracy of mass assignment can be validated by comparing spectra from two or more lap conditions, as previously reported.

## RESULTS AND DISCUSSION

**Figure 2:**
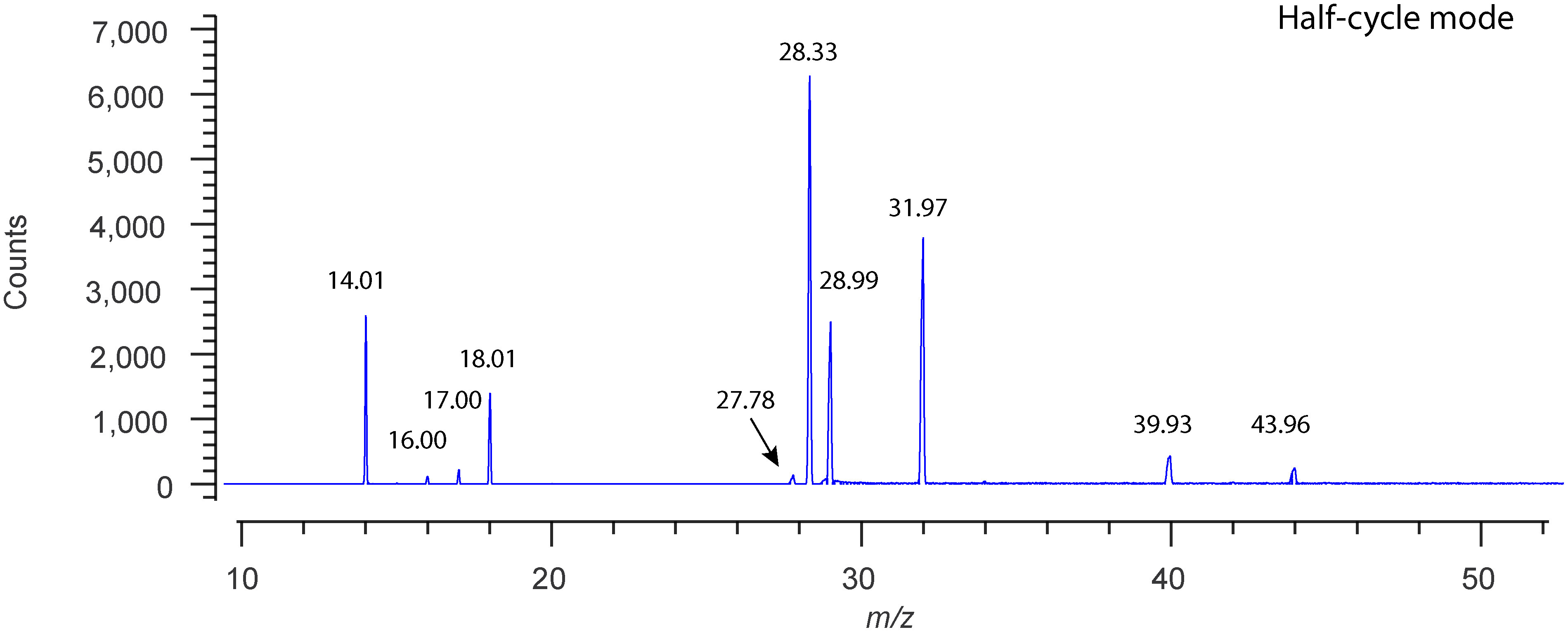
Fig. 2. Mass spectrum of a model gas mixture in the half-cycle mode.

Figure 2 shows the mass spectrum of the model gas mixture in the half-cycle mode using ion counting.^16)^ Since the sample gas was prepared in nitrogen, *m*/*z* 28 for nitrogen ion counting was over-scaled; however, the count rate for other ions was less than 37%, which is a good range for ion counting with a linear response for abundance. The half-cycle mode spectrum shows that the sample mixture contains at least eight peaks in an *m*/*z* range between 14 and 44.

**Figure 3:**
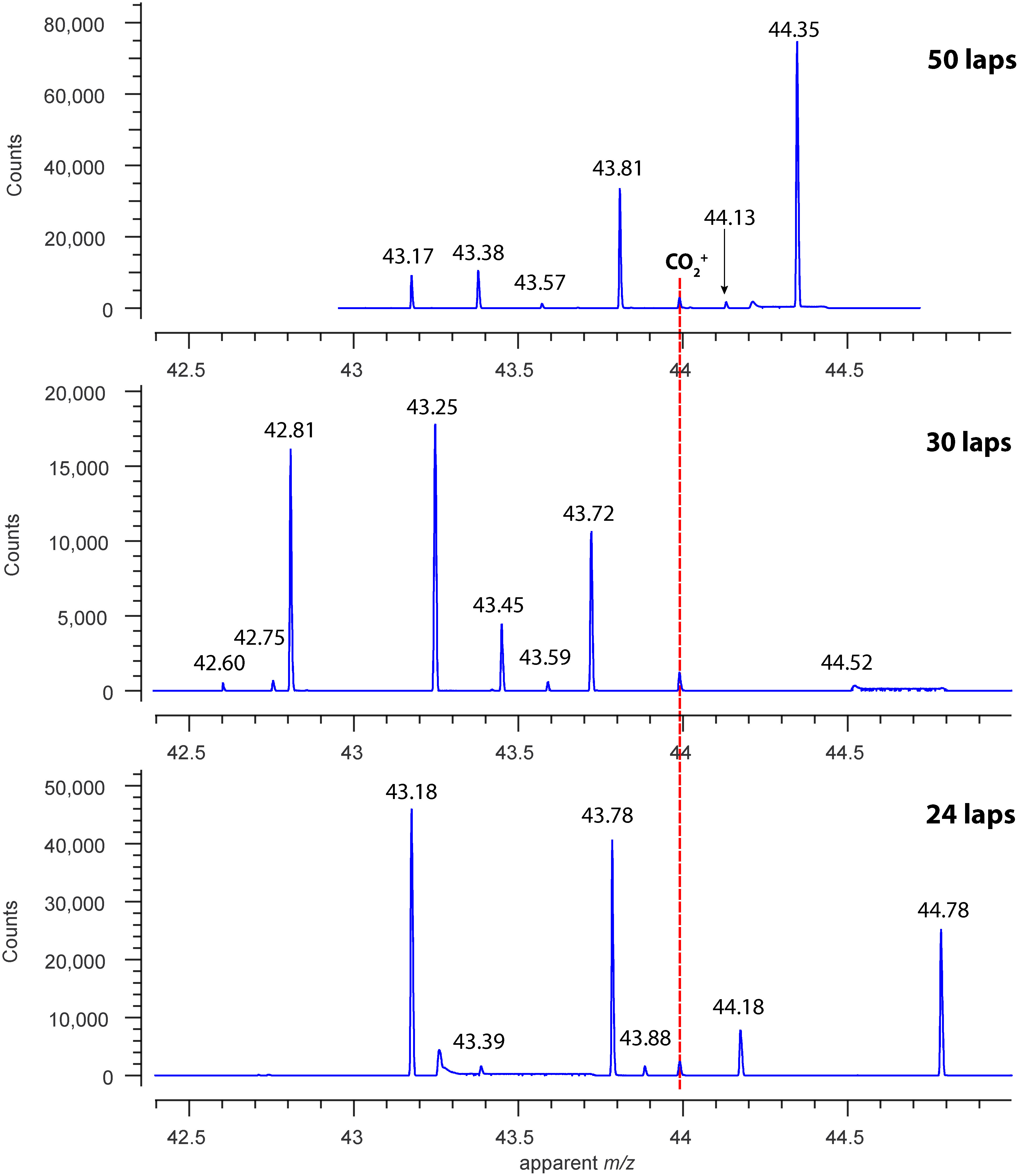
Fig. 3. Mass spectra of a model gas sample analyzed at 24, 30, and 50 laps for CO_2_^+^, which contain ion at different lap numbers.

Figure 3 shows the TOF spectra obtained at 24, 30, and 50 laps for an ion of *m*/*z* 44, which is the highest *m*/*z* in this sample. The vertical dashed-line spanning the three stacked spectra indicates the exact mass for CO_2_^+^, and the peak located on the dashed line was then identified and validated as a CO_2_^+^ ion. None of the other peaks that appeared in Fig. 3 matched between different lap conditions, thus indicating that the concurrent mass assignment for those peaks was not correct. The concurrently assigned mass shown on the top of each peak in Fig. 3 is an “apparent *m*/*z*.” Because the *m*/*z* range for the sample is known to be 14 and 44, the possible lap number for each ion must therefore be in the range of 24 to 43 for 24 laps condition (20 possibilities), 30 to 53 for 30 laps condition (24 possibilities), and 50 to 89 for 50 laps condition (40 possibilities). As described in the “Mass assignment algorithm” section, a list of all possible *m*/*z* values was calculated for each TOF value on the spectrum and was compared to the peak list obtained from half-cycle mode, as shown in Table 1. The lap number for a peak with an apparent *m*/*z* of 44.348 on the 50 laps condition spectrum has two candidates for lap-numbers of 59 and 62 as shown in Table 1, which corresponds to *m*/*z* 31.97 and 28.99 peaks on the half-cycle mode spectrum. Although these two peaks give the same TOF, which gives the same apparent *m*/*z* at 50 laps condition, they are clearly resolved by the 24 and 30 laps conditions.

The numbers shown on the spectra are apparent (observed) masses.

**Table 1:** Table 1. Peak list obtained from the analysis of the sample gas at the half-cycle mode, and *m*/*z*-matched ions observed in 24, 30, and 50 laps spectra.

TOF (μs)	*m*/*z*	Abundance (counts)	Apparent *m*/*z* on		
			24 laps	30 laps	50 laps
2.3201	14.01	10954	43.786 (43)	42.808 (53)	43.809 (89)
2.4635	16.00	592	43.387 (40)	43.590 (50)	43.572 (83)
2.5329	17.00	936	43.885 (39)	42.755 (48)	44.132 (81)
2.6001	18.01	6252	44.175 (38)	43.450 (47)	43.379 (78)
3.2370	28.99	13085	44.784 (30)	43.721 (37)	44.348 (62)*
3.3879	31.97	19814	43.176 (28)	43.247 (35)	44.348 (59)*
3.7592	39.93	2832	43.261 (25)	42.603 (31)	43.176 (52)
3.9333	43.96	1387	43.989 (24)	43.989 (30)	43.990 (50)

The lap number determined for each peak was written in the parentheses. A peak with an apparent *m*/*z* 44.35 on 50 laps condition can be matched for ions of *m*/*z* 28.99 (at 62 laps) and *m*/*z* 31.97 (at 59 laps).

All ions assigned as a pair of apparent *m*/*z* and lap number are listed in Table 2, which was recompiled from assigned *m*/*z* values from the results shown in Table 1. The assigned *m*/*z* can be validated from the *m*/*z* difference between spectra acquired at 24, 30, and 50 laps. For example, an ion assigned to *m*/*z* 14.002 in a 24-lap spectrum can also be found in two other spectra as *m*/*z* 14.002 and 14.003. An ion assigned to *m*/*z* 15.994 is identified on all three spectra with a value that matches up to three figures after the decimal point. Each peak was identified from the assigned *m*/*z*, and the compounds in the model sample and the mass errors for each assigned mass are also listed in Table 2.

**Table 2:** Table 2. Mass and peak assignment results for ion peaks.

CO_2_^+^ 24 laps						
Apparent *m*/*z*	Ion	Exact mass	Assigned mass	Error mDa	Lap	TOF μs
43.786	N^+^	14.0025	14.0021	−0.44	43	125.3506
43.387	O^+^	15.9944	15.9940	−0.34	40	124.7804
43.885	OH^+^	17.0022	17.0020	−0.19	39	125.4921
44.175	H_2_O^+^	18.0100	18.0099	−0.12	38	125.9064
44.784	^15^NN^+^	29.0026	29.0028	0.15	30	126.7699
43.176	O_2_^+^	31.9893	31.9897	0.46	28	124.4767
43.261	Ar^+^	39.9618	39.9635	1.68	25	124.5991
43.989	CO_2_^+^	43.9893	43.9899	0.60	30	156.0695
CO_2_^+^ 30 laps						
42.808	N^+^	14.0025	14.0022	−0.34	53	153.9621
43.590	O^+^	15.9944	15.9941	−0.30	50	155.3590
42.755	OH^+^	17.0022	17.0020	−0.14	48	153.8669
43.450	H_2_O^+^	18.0100	18.0099	−0.11	47	155.1099
43.721	^15^NN^+^	29.0026	29.0026	0.01	37	155.5935
43.247	O_2_^+^	31.9893	31.9896	0.35	35	154.7483
42.603	Ar^+^	39.9618	39.9599	−1.92	31	153.5936
43.989	CO_2_^+^	43.9893	43.9899	0.60	30	156.0695
CO_2_^+^ 50 laps						
43.809	N^+^	14.0025	14.0026	0.06	89	256.9657
43.572	O^+^	15.9944	15.9944	−0.01	83	256.2703
44.132	OH^+^	17.0022	17.0023	0.07	81	257.9086
43.379	H_2_O^+^	18.0100	18.0101	0.13	78	255.7014
44.348	^15^NN^+^	29.0026	29.0036	0.94	62	258.5387*
44.348	O_2_^+^	31.9893	31.9899	0.62	59	258.5387*
43.176	Ar^+^	39.9618	39.9628	1.01	52	255.1034
43.990	CO_2_^+^	43.9893	43.9903	0.98	50	257.4951

The *m*/*z* for seven peaks detected in the spectra of the standard gas sample acquired under conditions where CO_2_^+^ is 24, 30, and 50 laps were identified with less than half a milli-dalton of error, except for the argon ion. The argon ion was shown to be very close to the ejection sector open timing at the 30 and 50 laps condition, and, unfortunately, it was also affected by the excess N_2_^+^ ions at 24 laps. The Ar^+^ can be separated very well at a CO_2_^+^ 26 laps condition, where Ar^+^ peak will be 142 ns (12 times of peak width) away from the closest adjacent peak (41 laps of H_2_O^+^) listed in Table 2.

A possible false *m*/*z* assignment was further considered. A procedure for assigning *m*/*z* presented here is based on computing an array of *m* from experimentally obtained TOF (*t*) values using Eq. 4; and is compared to another array of *m*, which was acquired by yet another lap condition, by a different ejection sector open timing.

**Figure 4:**
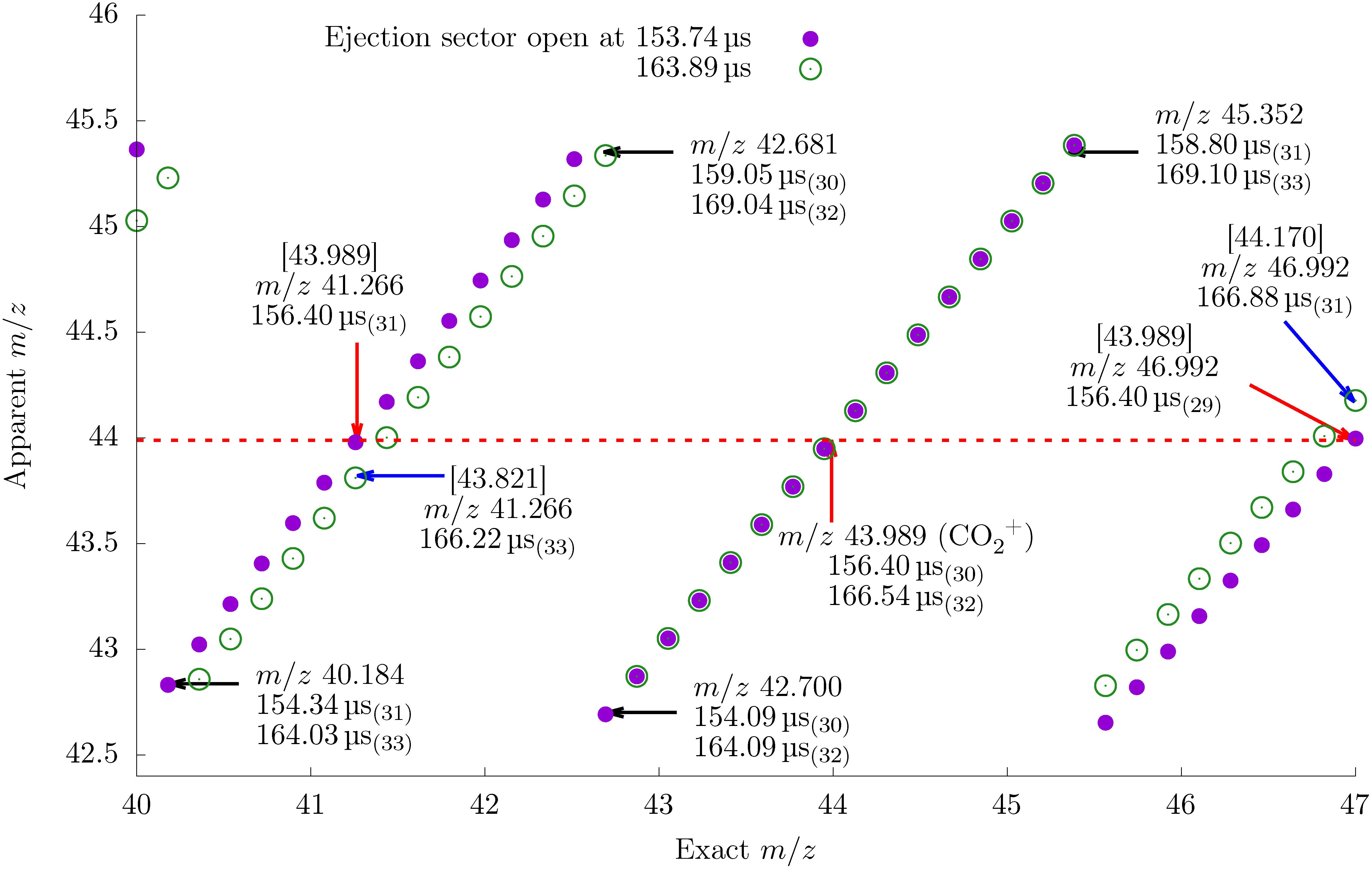
Fig. 4. Relationship between exact and apparent *m*/*z* at 153.74 μs (closed circle) and 163.89 μs (open circle) ejection sector open timing. Each point on the plot represents an ion every 0.175 Da. Opening the ejection sector at 153.74 and 163.89 μs results in 30 and 32 laps of CO_2_^+^ being detected at 156.40 and 166.54 μs on the TOF spectrum. The number in square brackets indicates the apparent *m*/*z*, and the number followed by *m*/*z* indicates the exact *m*/*z*.

Figure 4 shows a computed “apparent *m*/*z*” and exact *m*/*z* relationship for the *m*/*z* range between 40 and 47. For example, acquire TOF spectra by alternatively switching two ejection sector open timings for 153.73 and 163.89 μs, the ions in the *m*/*z* range 42.70 to 45.35 would be detected at 30 and 32-lap, respectively, in this condition. Assuming we have an ion peak on TOF 156.40 and 166.54 μs on both waveforms; the *m*/*z* can be assigned as 43.989 since the apparent *m*/*z* calculated on the assumption of a pair of 30 and 32-lap are both matched. Any ion appearing on 156.40 μs on a 30-lap condition waveform, and also a peak found at 166.22 μs on a 32-lap condition waveform can be assigned as *m*/*z* 41.266, since the apparent *m*/*z* calculated on the assumption of a pair of 31 and 33-lap are both matched. Curves representing an apparent *m*/*z* and exact *m*/*z* relationship are never overlayed between given lap conditions except for an *m*/*z* range that matches the correct lap number of the ion; therefore, the false *m*/*z* assign rate is significantly low.

## CONCLUSION

Two algorithms for identifying a pair of lap numbers and TOF from a mass spectrum acquired by a multi-turn TOF mass spectrometer are presented. The algorithms were evaluated using a model sample gas mixture and seven ions were successfully determined from different lap conditions on a lap-superimposed spectrum.

The first algorithm is applicable for target ion-oriented analyses, where the ions to be determined are known in advance; TOF and lap-number pair for each ion can be precisely calculated under the given analyzer conditions, such as 24 laps of CO_2_^+^. Using the calculated TOF, or the apparent *m*/*z*, individual ions can be identified from a mass spectrum, even when lap-superimposition is present. Peak assignments can be self-validated by comparison to a mass spectrum acquired under different lap conditions.

When an ion appeared at a lap number that was different from the other lap conditions, the TOF difference for the two identical peaks is the N-multiplication of an orbital period of the ion. Therefore, comparing two peaks acquired under different lap conditions and finding a pair of matching lap numbers to both TOF permit an ion for the corresponding peak to be determined. The most general method for determining *m*/*z* for an unknown peak involves comparing spectra acquired by the half-cycle mode with the multi-turn mode. By comparing a peak list of triplicate spectra and a peak list on a half-cycle mode spectrum, seven ions were successfully assigned with an error less than 0.6 mDa, except for Ar^+^, which was affected by the presence of excess nitrogen at 24 laps, and being too close to the ejection sector open timing at 30 and 50 laps. The ejection sector open timing for 30 and 50 laps conditions was set to 153.03 and 254.64 μs, respectively. The Ar^+^ takes 0.7 μs to pass through the ejection sector (0.063 m) under a given condition; therefore, Ar^+^ crosses the ejection sector that starts opening. As we previously reported,^6)^ during the opening and closing of the ejection sector, approximately 0.35 μs of time is available to select and quantitatively extract a monoisotopic ion; the effective TOF range that can be calculated from the *m*/*z* candidate list avoiding an error is 0.35 μs after the ejection sector open timing.

## Notes

Mass Spectrom (Tokyo) 2021; 10(1): A0098

## References

[1] H. Wollnik, M. Przewloka. Time-of-flight mass spectrometers with multiply reflected ion trajectories. *Int. J. Mass Spectrom. Ion Process.* 96: 267–274, 1990. doi: 10.1016/0168-1176(90)85127-N

[2] P. Schury, Y. Ito, M. Wada, H. Wollnik. Wide-band mass measurements with a multi-reflection time-of-flight mass spectrograph. *Int. J. Mass Spectrom.* 359: 19–25, 2014. doi: 10.1016/j.ijms.2013.11.005

[3] M. Toyoda, D. Okumura, M. Ishihara, I. Katakuse. Multi-turn time-of-flight mass spectrometers with electrostatic sectors. *J. Mass Spectrom.* 38: 1125–1142, 2003. doi: 10.1002/jms.546 14648820 10.1002/jms.546

[4] S. Shimma, H. Nagao, J. Aoki, K. Takahashi, S. Miki, M. Toyoda. Miniaturized high-resolution time-of-flight mass spectrometer MULTUM-S II with an infinite flight path. *Anal. Chem.* 82: 8456–8463, 2010. doi: 10.1021/ac1010348 20860354 10.1021/ac1010348

[5] T. Hondo, K. R. Jensen, J. Aoki, M. Toyoda. A new approach for accurate mass assignment on a multi-turn time-of-flight mass spectrometer. *Eur. J. Mass Spectrom.* (Chichester) 23: 385–392, 2017. doi: 10.1177/1469066717723755 29183186 10.1177/1469066717723755

[6] T. Hondo, H. Kobayashi, M. Toyoda. Selective extraction of a monoisotopic ion while keeping the other ions in flight on a multi-turn time-of-flight mass spectrometer. *Mass Spectrom.* (Tokyo) 9: A0088, 2020. doi: 10.5702/massspectrometry.A0088 32944489 10.5702/massspectrometry.A0088 PMC7471867

[7] T. Hondo, N. Nakayama, M. Toyoda. Gas chromatography/miniaturized time-of-flight mass spectrometry technique for high-throughput quantitative on-site field analysis. *Int. J. Mass Spectrom.* 463: 116555, 2021. doi: 10.1016/j.ijms.2021.116555

[8] P. Schury, M. Wada, Y. Ito, S. Naimi, T. Sonoda, H. Mita, A. Takamine, K. Okada, H. Wollnik, S. Chon, H. Haba, D. Kaji, H. Koura, H. Miyatake, K. Morimoto, K. Morita, A. Ozawa. A multi-reflection time-of-flight mass spectrograph for short-lived and super-heavy nuclei. *Nucl. Instrum. Methods Phys. Res. B* 317: 537–543, 2013. doi: 10.1016/j.nimb.2013.06.025

[9] P. Fischer, L. Schweikhard. Multiple-ion-ejection multi-reflection time-of-flight mass spectrometry for single-reference mass measurements with lapping ion species. *Rev. Sci. Instrum.* 91: 023201, 2020. doi: 10.1063/1.5131582 32113431 10.1063/1.5131582

[10] K. R. Jensen, T. Hondo, H. Sumino, M. Toyoda. Instrumentation and method development for on-site analysis of helium isotopes. *Anal. Chem.* 89: 7535–7540, 2017. doi: 10.1021/acs.analchem.7b01299 28631473 10.1021/acs.analchem.7b01299

[11] N. Nakayama, Y. Toma, Y. Iwai, H. Furutani, T. Hondo, R. Hatano, M. Toyoda. Mass spectrometric multiple soil-gas flux measurement system with a portable high-resolution mass spectrometer (MULTUM) coupled to an automatic chamber for continuous field observations. *Atmos. Meas. Tech.* 13: 6657–6673, 2020. doi: 10.5194/amt-13-6657-2020

[12] H. Kobayashi, T. Hondo, N. Imaoka, M. Suyama, M. Toyoda. Development of novel ion detector that combines a microchannel plate with an avalanche diode. *Nucl. Instrum. Methods Phys. Res. A* 971: 164110, 2020. doi: 10.1016/j.nima.2020.164110

[13] R. J. Cotter. in Time-of-Flight Mass Spectrometry: Instrumentation and Applications in Biological Research, American Chemical Society, 1997, pp. 20–21.

[14] H. Cohen, E. Porat. Fast set intersection and two-patterns matching. *Theor. Comput. Sci.* 411: 3795–3800, 2010. doi: 10.1016/j.tcs.2010.06.002

[15] D. E. Knuth. Optimum binary search trees. *Acta Inform.* 1: 14–25, 1971. doi: 10.1007/BF00264289

[16] Y. Kawai, T. Hondo, K. R. Jensen, M. Toyoda, K. Terada. Improved quantitative dynamic range of time-of-flight mass spectrometry by simultaneously waveform-averaging and ion-counting data acquisition. *J. Am. Soc. Mass Spectrom.* 29: 1403–1407, 2018. doi: 10.1007/s13361-018-1967-1 29700728 10.1007/s13361-018-1967-1

